# Probiotic engineering: towards development of robust probiotic strains with enhanced functional properties and for targeted control of enteric pathogens

**DOI:** 10.1186/s13099-017-0178-9

**Published:** 2017-05-08

**Authors:** Moloko Gloria Mathipa, Mapitsi Silvester Thantsha

**Affiliations:** Department of Microbiology and Plant Pathology, University of Pretoria, New Agricultural Sciences Building, Pretoria, 0002 South Africa

**Keywords:** Foodborne pathogens, Antibiotic resistance, Probiotics, Bioengineering

## Abstract

There is a growing concern about the increase in human morbidity and mortality caused by foodborne pathogens. Antibiotics were and still are used as the first line of defense against these pathogens, but an increase in the development of bacterial antibiotic resistance has led to a need for alternative effective interventions. Probiotics are used as dietary supplements to promote gut health and for prevention or alleviation of enteric infections. They are currently used as generics, thus making them non-specific for different pathogens. A good understanding of the infection cycle of the foodborne pathogens as well as the virulence factors involved in causing an infection can offer an alternative treatment with specificity. This specificity is attained through the bioengineering of probiotics, a process by which the specific gene of a pathogen is incorporated into the probiotic. Such a process will subsequently result in the inhibition of the pathogen and hence its infection. Recombinant probiotics offer an alternative novel therapeutic approach in the treatment of foodborne infections. This review article focuses on various strategies of bioengineered probiotics, their successes, failures and potential future prospects for their applications.

## Background

Poor hygiene and sanitation during food preparation can lead to the presence of different foodborne pathogens in food. Some of these pathogens or their toxins produced either before or after ingestion of such foods can either act locally within the gastrointestinal tract (GIT), leading to development of illnesses, or disseminate to other parts of the body and damage cells/tissues and ultimately the immune system [1]. Incidences of foodborne illnesses are high in most developing countries as food control is a low priority issue due to limited funds. As a result of this, food pathogens are the leading cause of illnesses and death in these countries [2]. Most foodborne illnesses cause diarrhoea, which is the primary symptom. Most societies consider diarrhoea a normal, natural condition; therefore, it goes unnoticed and/or untreated. Recently, the World Health Organization reported that of the 600 million global total cases of foodborne illness recorded in 2010, 550 million were due to infectious agents causing diarrhoea, of which 120 million and 96 million cases were caused by norovirus and *Campylobacter* spp., respectively. Diarrhoeal disease agents were responsible for approximately 55% (230,000 out of 420,000) deaths, with 59,000, 37,000, 35,000 and 26,000 deaths attributed to non-typhoidal *Salmonella enterica*, enteropathogenic *E. coli* (EPEC) and enterotoxigenic *E. coli* (ETEC), respectively [3]. These illnesses are not confined to developing countries. In the United States, foodborne pathogens cause an estimated 9.4 million illnesses, 55,961 hospitalizations and 1351 deaths each year [4]. Enteric pathogens account for high morbidity and mortality and are considered to be the fifth leading cause of death at all ages worldwide [5].

Probiotics have been used to restore the balance of the gut microbial ecosystem and control pathogenic infections. They are defined as “*live microorganisms that when administered in adequate amounts confer a health benefit on the host*” [6]. Their administration assists in the prevention and control of foodborne illnesses, through a number of mechanisms including but not limited to, competitive exclusion of pathogens in the GIT, modulation of the host immune system and strengthening of the intestinal barrier [7–9]. Although probiotics have proven successful in the control of enteric pathogens, they do have limitations. They are generic in nature and often fail to inhibit the attachment of certain pathogens at specific sites of infection and induce low levels of an immune response [10]. A thorough understanding of the limitations of conventional probiotics, the behaviour of the pathogens and the mechanisms by which they cause disease [11] provides possibilities to design new probiotic strains with desired characteristics and functionalities. Through genetic modification, novel bioengineered probiotic strains can be produced. Functioning of conventional probiotics in these novel strains can be strengthened to influence critical steps in pathogenesis. The strains can also be used to deliver drugs or vaccines, target a specific pathogen or toxin, mimic surface receptors and enhance an immune response within the host [12].

## Probiotics

Probiotics include mainly bacteria from the genera *Streptococcus, Enterococcus,* Pediococci*, Weissella* and *Lactococcus* [13] but the most common ones used belong to *Lactobacillus* and *Bifidobacteria* spp. These bacteria have met the criteria necessary to consider them as probiotics and they also have nutritional and therapeutic effects [14]. One of the criteria that bacteria must meet in order for them to be regarded as probiotics is that they have to be able to survive and thrive throughout the GIT conditions and confer their beneficial effects. It is therefore important to understand their mechanisms of action in order for them to be used both prophylactic and as treatment options for the different foodborne diseases. The presence of foodborne pathogens in the human GIT affects the balance of the “good to bad” microorganisms. Apart from the presence of the pathogens in the GIT, there are other factors that can affect the balance of the microorganisms in the host GIT. These factors include stress, illness or antibiotic treatment, which changes the balance in the GIT in favour of harmful bacteria [15, 16]. One of the characteristics of probiotics is that they are able to protect the host from microbial imbalance. Table 1 gives a summarized overview of the different mechanisms by which probiotics exclude pathogens from the human GIT, which are discussed in more detail below.

**Table 1:** Mechanisms of action of probiotics

Mechanism of action	Probiotic bacteria	Pathogen	Functionality	References
Competitive exclusion	*L. acidophilus*; *L. johnsonii*	*S. enterica* serovar Typhimurium, Enteropathogenic *E. coli* (EPEC), *Yersinia pseudotuberculosis, L. monocytogenes*	Inhibited adherence of pathogens to Caco-2 cells	[33]
	*L. casei* subsp*. rhamnosus*	EPEC, Enterotoxigenic *E. coli* (ETEC), *Klebsiella pneumoniae*	Inhibited adherence of pathogens to Caco-2 cells	[33]
	*L. rhamnosus; L. acidophilus*	*E. coli* O157:H7	Inhibited adherence of pathogens to T-84 epithelial cell; inhibited colonization to Caco-2 cells	[33, 51]
	*L. acidophilus*	*H. pylori*	Production of lacticins A164 and BH5	[37]
Production of inhibitory substances	*L. lactis*	*E. coli*, *Salmonella*	Production of alyteserin-1a and A3APO	[31]
	*B. longum*	*Clostridium difficile, E. coli*	Production of bacteriocin	[35]
	*L. salivarius*	*L. monocytogenes*	Production of bacteriocin Abp118	[35]
	*L. sake*	*L. monocytogenes*	Production of bacteriocin sakacin A	[39]
	*L. plantarum*	*L. monocytogenes*	Production of bacteriocins (plantaricins)	[40]
	*L. lactis*	*C. difficile*	Production of lacticin 3147	[45]
	*L. lactis, L. casei, L. acidophilus*	*E. coli* O157:H7	Reduced the growth of pathogen by lactic acid production and pH reductive effect	[33]
Immune system modulation	*L. reuteri*	*Salmonella*	Increased the production anti-*Salmonella* IgM	[35]
	*L. rhamnosus*	*E. coli* O157:H7	Increased intestinal anti-*E. coli* IgA responses and blood leukocyte phagocytic activity	[33]
	*L. acidophilus*, *L. rhamnosus* GG, *L. johnsonii* La1	*S.* Typhimurium	Increased the production of anti-*Salmonella* IgA	[42, 43]
Improved barrier function	*L. plantarum*, *L. rhamnosus* GG	*E. coli* O157:H7	Subvert the adherence of pathogen by increasing MUC2 and MUC3 in HT-29 epithelial cell line	[45, 47]
	*L. acidophilus*	*E. coli*	Protected against F-actin rearrangement, which was induced in an epithelial cell line on exposure to a pathogenic *E. coli*	[45]
	*S. thermophilus; L. acidophilus*	Enteroinvasive *E. coli* (EIEC)	Increased transepithelial resistance, maintenance and enhancement of cytoskeletal and tight junctional protein phosphorylation in HT29 and Caco-2 cell lines	[60]

### Probiotics’ mechanisms of action against enteric pathogens

#### Competitive exclusion

Probiotics can exclude or reduce the growth of other microorganisms in the GIT either through competition for nutrients or adherence space [17–19]. Microorganisms in any environment require nutrients to multiply and either cause or alleviate infections. The GIT is well known for its abundance in nutrients, making it a suitable environment for microbial colonization. The potential of probiotics to outcompete pathogens for these nutrients favours their growth over that of the pathogens [20]. During competition for nutrients, probiotics produce metabolites such as volatile fatty acids reducing the pH of the GIT. The reduction in the pH of the GIT makes it an unfriendly environment for pathogens and will thus lead to their inhibition because most of them cannot grow at low pH [21, 22].

Competition for adherence space refers to the situation when the presence of probiotics blocks pathogenic bacteria from colonizing favourite sites such as the intestinal villi, goblet cells and the colonic crypts [22]. Attachment to the surfaces of intestinal epithelial cells is a key pathogenic factor of enteric pathogens [23]. At the same time, colonization resistance, through which attachment and multiplication of the pathogens on the intestinal mucosal membrane is prohibited, is a critical function of the microbiota [24, 25]. Probiotics bind to intestinal cells via electrostatic interactions, steric forces or specific surface proteins. This affords them the ability to bind to these cells in high quantities [17, 26], thereby physically blocking the sites, leaving no space for the pathogens to adhere and subsequently cause infection [27].

Probiotic LAB have a greater ability to adhere to the epithelial cells than pathogens [28]. Lactobacilli and bifidobacteria share carbohydrate-binding specificities with some enteropathogens [29]. *Bifidobacterium bifidum* and *Lactobacillus reuteri* bind to glycolipids on the surface of the host cells to prevent attachment of certain pathogens that also bind to specific surface glycolipids [8]. Thirabunyanon and Thongwittaya [30] reported in their study that they observed a reduction in attachment of *Salmonella enteritidis* to the surfaces of intestinal epithelial cells in the presence of probiotic *Bacillus subtilis* NC11. This led to a complete exclusion of this pathogen in the GIT, which is the site where the infection process is initiated. This was corroborated with poor survival of the pathogen attributed to limited nutrients for their growth and proliferation, and unavailability of adherent space in the GIT.

#### Production of inhibitory substances

In order to gain a competitive advantage when competing for space and nutrients, microorganisms release antimicrobial compounds. Antimicrobial compounds have a direct inhibition on several target pathogens [31]. The mechanisms used by probiotics to inhibit pathogenic bacteria are interconnected. As already mentioned, exclusion of pathogens occurs due to the ability of probiotics to secrete organic acids such as acetic and lactic acids [32]. The production of these organic acids leads to a decrease in the pH of the environment, making the microenvironment acidic thereby excluding pathogens that cannot survive acidic conditions [8]. The organic acids also have an effect on the pathogen metabolism and production of toxins, ultimately preventing disease.

The anti-pathogenic activity of probiotics is multifactorial [33]. In addition to the acids mentioned above, probiotics can produce other metabolites with antibacterial properties, such as H_2_O_2_ and bacteriocins, also referred to as non-lactic acid molecules [33–36, 41]. Bacteriocins are small antimicrobial peptides produced for bacterial competition in a natural ecosystem [31]. They may act as colonizing peptides by facilitating the introduction of probiotics into an already occupied niche on the intestinal epithelium. This competitive advantage allows for an increase in the density of probiotic bacteria on the surface of the host intestines [36]. They can also act as killing peptides, by directly affecting pathogens. A study by Kim et al. [37] evaluated the antimicrobial activity of the bacteriocins: lacticin, pediocin and leucocin, produced by lactic acid bacteria against *Helicobacter pylori.* These bacteriocins were able to significantly inhibit the growth of *H. pylori,* with lacticin having the most inhibitory effect against this gut pathogen.

*Lactobacillus acidophilus* has been reported to produce metabolites such as acidophilin, lactocidin and acidolin [35], whereas bifidobacteria produces bacteriocin-like substances [38], all inhibiting bacteria such as *Bacillus*, *Salmonella*, *Staphylococcus* and *E. coli*, *Clostridium perfringens*, *Listeria* species, among others [35, 39, 40]. Fayol-Messaoudi et al. [41] reported that the antibacterial effects of the probiotic *Lactobacillus* that inhibited the growth and resulted in pathogen death were due to the synergistic action of lactic acid and the secreted non-lactic acid molecules. Certain probiotic strains can also stimulate the increase in the expression of host cell antimicrobial peptides. The intestinal cells of the host are able to produce defensins which can inhibit the functioning of pathogens thus aiding in the protection of the intestinal barrier [36].

#### Immune system modulation

Probiotics can displace pathogens through stimulation of host immunity [42]. There is considerable evidence to support the notion that probiotics displace pathogens in the GIT through stimulation of specific and non-specific immunity to inhibit bacteria causing intestinal diseases [43, 44]. They modulate the host’s immune system against the pathogens harmful antigens by the activation of lymphocytes and production of antibodies [45]. They can also stimulate the effects of different cells involved in innate and adaptive immunity, such as dendritic cells, macrophages, T cells and B cells, which enhances phagocytosis of gut pathogens [46]. Probiotic strains such as *Lactobacillus rhamnosus* and *Lactobacillus plantarum* adhere to gut-associated lymphoid tissue enhancing both systemic and mucosal immunity [9]. These probiotics strains enhance immunity by up-regulating production of intestinal mucins (MUC2 and MUC3), which disrupts the adherence of pathogens to the intestinal epithelium, consequently preventing pathogen translocation. Furthermore, they induce expression of TGFβ and interleukins (IL-10 and IL-6) by epithelial cells, which enhances production and secretion of IgA [47].

Probiotics can be recognized by the immune system through pattern recognition molecules such as Toll-like receptors. This recognition can lead to various intracellular signal transduction cascades and enhancement or reduction of pro and anti-inflammatory cytokines. There are different studies supporting this evidence. In 2000, Fang et al. [43] divided 30 healthy volunteers into three different treatment groups, with each group consuming *Lactobacillus GG*, *Lactococcus lactis* and placebo (ethyl cellulose), respectively, for 7 days. All the treatment groups were given an attenuated *Salmonella typhi* Ty21a oral vaccine. The results showed that there was an increase in the humoral immune response in the treatment group receiving the probiotics as compared to the control group. Probiotics are able to stimulate the production of antibodies in the intestinal lumen, specifically immunoglobulin A. Immunoglobulin A (IgA) represents the first-line defense against infection and can inhibit the adhesion of pathogenic bacteria to the intestinal epithelia. It can interfere with adhesive cell receptors on the pathogen’s cell surface and cause bacterial agglutination. One study indicated that oral administration of *Lactobacillus casei* enhanced the concentration of IgA in infants suffering from diarrhoea, thereby shortening the duration of this symptom [46, 48]. Ng et al. [45] reported that administration of *L. rhamnosus* resulted in enhanced non-specific humoral responses reflected by an increase in the levels of circulating IgG, IgA and IgM in children with acute gastroenteritis.

In addition to the above, probiotics can stimulate an anti-inflammatory response, which can be used as an approach to reduce inflammation caused by gastroenteritis, enterocolitis and irritable bowel syndrome [9]. An anti-inflammatory response is triggered when strains stimulate the activation of dendritic cells which secrete interleukin 10 (IL-10), a cytokine that plays a role in reducing inflammation. They also cause a decrease in the levels of proinflammatory cytokines during inflammation [46].

#### Improved barrier function

The integrity of the intestinal barrier needs to be maintained in order to prevent pathogens from reaching the intestinal cells, thereby leading to local and systemic infections. Gut pathogens have the ability to disrupt the barrier when there is an imbalance in the microbial gut ecosystem [49]. It has previously been reported that consumption of probiotics can maintain the barrier function and mucosal integrity, prevent chronic inflammation, thereby protecting the host against infections [50]. Probiotics decrease paracellular permeability, providing innate defense against pathogens and enhancing the physical impediment of the mucous layer [51]. They are also able to repair this barrier after damage that may have been caused by gut pathogens. As an approach to repair the intestinal barrier, probiotics can stimulate mucous secretion, chloride and water secretion and the binding together of submucosa cells by tight junctional proteins [8].

Goblet cells express rod-shaped mucins (MUCs), which are either localized to the cell membrane or secreted into the lumen to form the mucous layer [52, 53]. There are 18 mucin-type glycoproteins that are expressed by humans [49]. In the human intestinal cell lines, *Lactobacillus* species increased mucin expression (MUC2 by Caco-2 cells; MUC2 and MUC3 by HT29), thus blocking cellular adhesion and invasion by pathogenic *E. coli* [54, 55]. Madsen et al. [56] showed that the treatment of IL-10 gene-deficient mice with a combination probiotic VSL#3 (*L. casei, L. plantarum, L. acidophilus, L. delbrueckii* subsp. *bulgaricus, Bifidobacterium longum, B. breve, B. infantis, and Streptococcus salivarius* subsp. *thermophilus*), resulted in normalization of colonic physiologic function and barrier integrity leading to significant improvement in histologic disease [57].

Tight junctions (TJ) form the continuous intercellular barrier between epithelial cells, which is required to separate tissue spaces and regulate selective movement of solutes across the epithelium [58]. There are different proteins expressed on the TJ and the disruption of their expression leads to a dysfunctional epithelial barrier [57]. A study by Qin et al. [59] reported that *L. acidophilus* increases the expression of occludin, a major component of TJ, in the gut mucosa of animals with cecal ligation and perforation, leading to a reduced bacterial translocation. A different study by Resta-Lenert and Barrett [60] reported that probiotic bacteria, specifically *S. thermophilus* and *L. acidophilus*, prevented reduction in the enteroinvasive *E. coli*-induced phosphorylation of the proteins occludin and zonula occludens 1 (ZO-1), thereby preserving the TJ structure. Furthermore, Parassol et al. [61] showed that *L. casei* prevents the redistribution of the TJ protein ZO-1 away from the cell–cell contacts caused by infection with enteropathogenic *E. coli*.

## The use of conventional probiotics for control of selected food pathogens

Due to the widespread use of antibiotics as therapeutic agents and the misuse of these antibiotics, there has been an increase in the antibiotic resistance of bacteria, an imbalance of normal microflora and the presence of drug residues in food products [41]. This brought about a requirement for new intervention in the treatment of bacterial pathogens, leading to an escalation in the research field of the beneficial microorganisms, i.e. probiotics. Prevention and treatment of infections caused by the different pathogens is one of the reasons why probiotics extensively studied [62]. When studying the prevention and treatment of pathogens, it is important to consider the complexity of the intestinal environment where a network of interactions among the microorganisms of the resident microbiota, epithelial and immune cells associated with the GIT, and nutrients exist [63, 64]. The epithelial and the immune cells play a role in the modulation of the immune functions and they provide the first line of defense against the pathogenic bacteria. The resident microbiota have the ability to influence the composition and activity of the gut microbiota [62]. They also play a beneficial role in the treatment of disease caused by foodborne pathogens [65, 66]. Different microorganisms infect different parts of the host GIT, for example, *H. pylori*, infects the gastric and duodenal mucosa, *Salmonella* spp. and *Clostridium difficile* cause inflammation in ileum and colon, while *Shigella* sp. clearly prefers the colonic mucosa [67].

Previous studies have shown the effects of probiotics, that when consumed as part of the daily diet, they can maintain the immune system in an active state and prevent different intestinal disorders [62]. Valdez et al. [68] reported that certain LAB probiotics inhibit apoptosis of macrophage infected with *Salmonella* preventing salmonellosis. Cano and Perdigón [69] studied the preventative measure of *L. casei* CRL 431 against *S.* serovar Typhimurium, reporting that administrating probiotics prevented *S.* serovar Typhimurium infection (100% protection) after 14 days of the re-nutrition diet in mouse models. Findings of their study were confirmed by a different study [62], where the preventative and continuous administration of probiotic *L. casei* CRL 431 against *S.* serovar Typhimurium in a mouse model was studied. They reported that the study group fed the probiotic for 7 days before the introduction of the pathogen and post infection experienced less severe infection compared to the control group which did not consume probiotics. They furthermore reported that 7-day administration of probiotics post infection resulted in better protection against *Salmonella* infection. They concluded that the continuous administration of the probiotic diminished counts of the pathogens in the intestine as well as their spread outside this organ.

More studies have been conducted on different pathogens to show the efficacy of probiotic strains. *H. pylori* is a bacterium that plays a crucial role in the pathogenesis of chronic active gastritis and peptic ulcer disease in both adults and children [70] with increasing amount of evidence supporting the hypothesis that it is an important co-factor in the development of gastric cancer [71]. *H. pylori* has been linked to cancer; however, there is no vaccine licensed to prevent infection with this organism [72]. There are different therapeutic approaches that are used to treat *H. pylori*, including but not limited to the commonly used triple therapy with proton pump inhibitor (PPI), clarithromycin and either amoxicillin or metronidazole or dual-therapy high-dosage amoxicillin and PPI; however, there have been reports that suggest that some patients still remain infected after administration of these treatment [73]. Administration of alternative compounds that may increase the efficacy of the treatment and/or reduce side-effects is of particular interest [72]. There is growing evidence from different studies emphasizing the efficacy of probiotics in the management of *H. pylori* infection targeting different aspects of this infectious disease [74, 75]. Cats et al. [74] investigated whether readily available commercial preparation containing *L. casei* inhibits the growth of *H. pylori* in vitro. They reported that in vitro *L. casei* inhibits the growth of *H. pylori*; however, the probiotic cells have to be viable. In a different study, Bernet-Camard et al. [76] reported that probiotics such as *L. johnsonii* La1 (La1) or *L. rhamnosus* GG exert bacteriostatic or bactericidal activities against a wide range of pathogens, including *H. pylori*. Cruchet et al. [77] studied if the regular ingestion of a dietary product containing *L. johnsonii* La1 or *L. paracasei* ST11 would interfere with *H. pylori* colonization in children. They concluded that regular ingestion of the dietary product containing *L. johnsonii* La1 may represent an interesting alternative to modulate *H. pylori* colonization in children infected by this pathogen. Tursi et al. [78] demonstrated that a 10-day quadruple anti-helicobacter therapy with ranitidine bismuth citrate (RBC) plus proton pump inhibitors (PPI), amoxicillin and tinidazole obtains a high eradication rate, whereas supplementation with *L. casei* significantly increased the eradication rate of *H. pylori* infection. This study concluded that the supplementation of the therapy with the administration of probiotics showed a slight improvement in the eradication of *H. pylori.* Probiotics can therefore be used as first course of anti-*H. pylori* treatment or can be used in conjugation with the first-line therapeutic approaches.

*Shigella* is an antibiotic-resistant bacterium [79, 80] that has been reported to cause gastroenteritis-induced deaths in 3–5 million children aged less than 5 years in developing countries [81, 82]. The emergence of multiple drug resistance to cost-effective antimicrobials against *Shigella* is a matter of concern in developing countries, and resistance pattern of this bacterium is the cause of numerous clinical problems worldwide [83]. Due to increased prevalence of its antibiotic resistance, the need for alternative treatment has therefore been deemed necessary. Zhang et al. [84] studied the antimicrobial activity of the probiotics *L. paracasei* subsp. *paracasei* M5-L, *L. rhamnosus* J10-L, *L. casei* Q8-L and *L. rhamnosus* GG (LGG) against *Shigella sonnei*. They reported that the tested lactobacilii strains showed strong antimicrobial activity against *S. sonnei*. In a study to screen for the antimicrobial activity of probiotics against *S. sonnei*, Zhang et al. [85] reported that *L. johnsonii* F0421 exhibited significant inhibitory activity and excluded, competed and displaced *S. sonnei* adhered to HT-29 cells. In a different study, Mirnejad et al. [83] evaluated the nature of antimicrobial substances and properties of *L. casei* against multi-drug-resistant clinical isolates of *S. flexneri* and *S. sonnei*. Their results indicated that *L. casei* showed strong antimicrobial activity against *S. flexneri* and *S. sonnei,* and they attributed pathogen inhibition to production of organic acids by the test *Lactobacillus*. In another study, Zou et al. [86] studied the antimicrobial activity of nisin, a bacteriocin produced by *L. lactis* strains, against *L. monocytogenes*, *Staphylococcus aureus*, *Salmonella typhimurium* and *Shigella boydii*. They reported that there was a decline in pathogen populations, which was ascribed to the changes in the fatty acid profiles, cell viability, membrane permeability and depolarization activity in response to nisin.

*Listeria monocytogenes* is a foodborne pathogen that causes devastating effects in the human host, causing disease conditions ranging from premature delivery and stillbirth in perinatal cases [87] to meningitis and septicemia in adults [88, 89]. There have been many studies using different probiotics to combat this food pathogen. In a study to demonstrate the activity of the antibacterial substances produced by bifidobacterial isolates, Touré et al. [90] isolated six infant bifidobacterial strains from breast-fed infant faeces, with a potential antimicrobial activity against *L. monocytogenes*. These isolates actively inhibited *L. monocytogenes* by producing a heat-stable proteinaceous substance. Their study indicated that the use of bifidobacterial strains capable of competing with pathogenic organisms following the probiotic approach would advantageously improve intestinal bacterial ecology and provides a useful alternative strategy for inhibiting intestinal pathogens. In 2007, Corr et al. [91] studied the pretreatment of C2Bbe1 cells, a clone of the Caco-2 human adenocarcinoma cell line with strains of *Bifidobacterium* and *Lactobacillus* to demonstrate that this can significantly interfere with subsequent invasion by *L. monocytogenes*. They reported that the pretreatment of intestinal epithelial cells with probiotic bacteria prior to infection with *L. monocytogenes* EGDe resulted in a significant decrease in listerial invasion (60–90%). In yet another study testing for the antagonistic effect of *Lactobacillus* strains against *E. coli* and *L. monocytogenes*, it was reported that *L. plantarum* WS4174 exhibited a stronger inhibitory effect against the Gram-positive *L. monocytogenes* LMO26, possibly due to the accumulation of lactic acid and higher sensitivity of *L. monocytogenes* to low pH [92].

## Limitations of conventional probiotics

Although probiotics provide numerous benefits to the host, they do have certain limitations. Certain studies have provided evidence that probiotic strains may be inefficient or ineffective in response to specific gut pathogens. Probiotics may release antimicrobial compounds that have a broad antimicrobial spectrum; however, reports have suggested that there are limitations in the success of probiotics targeting specific pathogens. Therefore, a cocktail of various probiotic strains would need to be produced in order to enhance the effects against different pathogens within the gut [93].

Contrary to earlier reports that probiotics exhibited inhibitory effect against *L*. *monocytogenes* [90, 91], according to Koo et al. [94], probiotics have a limited success in preventing the attachment of *L. monocytogenes* to intestinal monolayers. In their study, which used three experimental approaches of competitive exclusion, inhibition of adhesion or displacement, to determine whether selected lactobacilli would reduce adhesion of *L*. *monocytogenes* to Caco-2 cells, they showed that the percentages of *L*. *monocytogenes* adhesion in the presence and absence of probiotics were fairly similar. None of the lactobacilli and other LAB were able to significantly reduce adhesion or colonization on epithelial cells, even at higher numbers. Furthermore, an increase in the concentration of the probiotic strain also failed to displace the attached *L. monocytogenes.* The data from the study indicated the conventional LAB strains could not prevent adhesion of this pathogen.

Another report indicated that probiotics may also stimulate low levels of an immune response and low levels of an anti-inflammatory response [10]. *L. salivarius* and *B. infantis* were orally administered to mice suffering from colitis. Results indicated that TGF-β levels in mice treated and untreated with probiotics remained the same. TGF-β is an anti-inflammatory cytokine, and the levels of this cytokine were not significantly increased but still maintained by *L. salivarius;* however, these were not maintained in the presence of *B. infantis*.

Most probiotics are administered as food or capsules; therefore, they have to be able to withstand both the technological and gastrointestinal stress factors. The broad mode of action of probiotics and the differences from one probiotic to another is also an obstacle in their efficacy. It has been reported that the beneficial attributes of one strain or a cocktail of strains may not be reproducible and may vary from person to person [95]. In addition to that, the strain of the probiotic, the dosage, the route of administration, and the formulation of probiotic preparation can also affect their efficacy [94]. Taking these studies into consideration, it is evident that probiotics are still non-specific and non-discriminatory in their mode of action or ineffective in certain hosts [96].

The limitations discussed above introduce the need for more novel and innovative approaches in the use of probiotics for the prevention and treatment of foodborne pathogens. Previous literature has reported that the use of probiotics has been extended to deliver therapeutic and prophylactic molecules to the mucosal barrier of the host [94, 97, 98]. However, for that to be done successfully, a thorough understanding of the behaviour of the pathogens and their disease mechanisms is needed [11]. Such knowledge can then be used to increase the efficacy of the probiotics and later use of a specific probiotic for a specific pathogen or toxin. Thus, novel probiotic strains with enhanced or even targeted probiotic functioning can be produced. Bioengineering techniques offers an opportunity for the design of such recombinant probiotic strains.

## The concept of probiotic bioengineering or recombinant probiotics

The performance of the existing probiotic strains can be improved through the use of bioengineering. Bioengineering refers to the manipulation of a gene of a probiotic strain in order to improve the tolerance to the technological stress, including but not limited to temperature extremes, oxygen and acidification, during food production, and/or survival of the probiotic in the GIT, to confer beneficial effects to the host [99]. This strategy can be used in the design and construction of new probiotic strains harbouring genes of interest derived from the pathogens. It allows for the production of proteins that were initially not present within the microorganism. Virulence factors of the pathogens can be cloned and expressed into the probiotic strain and subsequent administration of the resultant recombinant probiotic strain will inhibit the development of infection and yield no clinical presentation of the symptoms. Furthermore, recombinant probiotics can be used to deliver drugs or vaccines, target specific pathogens or toxins, enhance an immune response and mimic cell surface receptors [100]. Most human receptors recognized by enteric pathogens or their toxins are well characterized. Also, by targeting a specific pathogen, this strategy deems the development of resistance to the vaccine or treatment unlikely. Bioengineering of probiotics is not entirely a new field, various researchers have reported on the successes attained with the use of such probiotics (Table 2). Culligan et al. [49] reported on the main advantages of using recombinant probiotics in the treatment of enteric infection. The next section of this review focuses on studies that were conducted on bioengineered probiotics aimed at improving different functional properties of the conventional strains.

**Table 2:** Applications of bioengineering

Applications	Probiotics	Genes/receptors expressed	Action	References
Improvement of stress tolerance	*L. paracasei*	Heat shock protein chaperones (GroES and GroEL)	Improved thermotolerance (heat tolerance) of probiotic; increased solvent resistance by the probiotic strain	[104]
	*L. salivarius*	Listerial betaine uptake system (BetL)	Increase in the resistance of the probiotic to several stresses	[110]
	*L. lactis*	Trehalose synthesis gene (*ostAB*)	Enhanced probiotic’s resistance to gastric acid protection of the probiotic against damage caused by acid, cold, or heat shock	[114, 115]
Production of antimicrobial peptides	*L. lactis*	A3APO and alyteserin	Successfully inhibited *E. coli* and *Salmonella*	[31]
	Probiotic *E. coli*	Cell receptor (ganglioside) for cholera toxin or ETEC heat-labile toxin	Enterotoxins are sequestered by the probiotic *E. coli* thus protecting host against diarrheal infection	[12]
	*L. reuteri*	Heat-stable (ST) and heat-labile (LT) enterotoxins	Successfully bound to the enterotoxins and prevented enterotoxicity in a mouse model	[12]
Enhancement of anti-inflammatory response	*L. lactis*	Elafin	Significant reduction in inflammation	[118]
	*L. lactis*	TGF-β	Overall reduction of inflammation and colitis	[120]
	*L. lactis*	IL-10	Successfully prevented colitis in murine models	[121]
	*L. lactis*	Anti-TNF-α nanobodies	Reduced the colonic inflammation	[123]
	*L. lactis*	Internalin A	Enhanced efficient internalization of *L. lactis* in the human intestinal cell line Caco-2	[124]
Enhancement of colonization exclusion	*L. paracasei*	*Listeria* adhesion protein (LAP)	Inhibited the adhesion of *Listeria* to host cells	[94]
	*L. lactis*	Surface-associated flagellin	Inhibited the binding and adhesion of pathogenic *E. coli* and *S. enterica*	[126]
	*L. acidophilus*	K99 fimbriae	Reduced the attachment of ETEC to porcine intestinal brush border	[129]
Receptor mimicry system and toxin neutralization	*E. coli* Nissle 1917; *L. lactis*	Galactosyl-transferase genes; Tetanus toxin fragment C (TTFC)	Recombinant bacteria neutralized shiga toxins, Stx1 or Stx2	[132]
			Increased IgA levels led to protection of the host against the infections of the mucous membrane	[135, 136]
	*E. coli* Nissle 1917	Receptor GM1	Protected infant mice from challenge with virulent *V. cholerae*	[139]
	*E. coli* Nissle 1917; *L. casei*	AI-2 co-expressed CAI-1	80% reduction in Ctx binding to the intestines of mice which reduced numbers of *V. cholerae* in treated mouse intestines	[140]
		Adhesins K99	Protected 80% of the vaccinated mice after challenge with a lethal dose of strains of ETEC K99 and K88	[142]
Vaccination	*L. lactis*	Virus spike protein VP8	Provided 100% protection against rotavirus infection	[145]

### Applications of probiotic bioengineering

#### Improvement of stress tolerance

There has been an increase in the use of probiotics due to their known effects to confer beneficial health to the host. However, there are still problems frequently associated with the incorporation of probiotic strains into food products. These problems include but are not limited to poor temperature, salt and oxygen tolerance of some species or strains. Different approaches including pre-adaptation to stress, the use of oxygen-impermeable containers, microencapsulation [101], incorporation of nutrients, and selection of stress-resistant strains have been used in an attempt to address these problems [102]. The use of bioengineering has been used in the field of stress adaptation, and there have been promising results.

The ability to confer additional stress tolerance in stress-sensitive cultures can lead to the development and delivery of novel probiotics with maximal therapeutic efficacy [103]. It has been reported that the two major heat shock proteins, GroES and GroEL, are essential for the survival of bacteria at all temperatures [104]. In a study by Desmond et al. [101], the effect of overexpression of these heat shock protein chaperones (GroES and GroEL) in the probiotic *L. paracasei* NFBC338 was investigated. Expression of these genes resulted in improved thermotolerance (heat tolerance) as well as increased solvent resistance by the probiotic strain. Furthermore, they compared the survival of the non-adapted parent strain, stress adapted and the recombinant probiotic during exposure to heat stress. They reported that the recombinant probiotic survived 10- and 54-fold better than the stress-adapted and non-adapted parent strains, respectively.

The survival of pathogens is usually dependent on the different systems that can help them overcome the different stress conditions present in the GIT. Three transport systems have to date been identified in *L. monocytogenes* that have been linked to betaine and carnitine uptake [105, 106]. The first of these is a gene encoding the secondary glycine betaine transporter, listerial betaine uptake system (BetL), which is linked to salt tolerance of *Listeria* [107, 108]. It has been reported that disrupting BetL results in reduced growth at 37 °C in complex media of elevated osmolarity [107]. The reduction in the initial betaine uptake in the absence of BetL leads to diminished intracellular solute pools [106], causing changes in the cell volume, intracellular solute concentration and the turgor pressure [109]. Sheehan et al. [110] studied the heterologous expression of the BetL into the probiotic strain *L. salivarius* UCC118 using a nisin-controlled expression system. They reported that expression of BetL led to an increase in the resistance of the probiotic to several stresses (osmo, cryo, baro and chill), spray- and freeze-drying. Later in another study these researchers demonstrated that *B. breve* UCC2003 harbouring the betaine uptake (BetL) gene displayed an improved tolerance to gastric juice and elevated osmolarity [111].

Trehalose is a non-reducing disaccharide ubiquitously distributed in nature and is well known for its role in protecting cells against a variety of stresses [112]. In *E. coli,* it is synthesized in response to high osmolarity [113]. Termont et al. [114] cloned the trehalose synthesis gene (*ostAB*) from *E. coli* into *L. lactis* and reported that there was an enhanced probiotic’s survival during freeze-drying, in high bile concentrations and its resistance to gastric acid. In a different study, Carvalho et al. [115] studied the expression of the trehalose synthesis in the same probiotic *L. lactis* and reported that trehalose plays a definite role in the protection of this bacterium against damage caused by acid, cold or heat shock. These studies provide evidence that expression of genes from pathogenic species to improve stress tolerance of probiotics has been explored with promising results. However, further scientific assessment is still required to analyse the benefit of using these genes and interpretation by risk–benefit analysis [103].

#### Production of antimicrobial peptides

The rise in development of antibiotic resistance of pathogens has led to a dire need for alternative methods to treat infections. Antimicrobial peptides (AMPs) have been explored as an alternative method for effective control of multi-drug resistant (MDR) pathogens [116]. As already mentioned, some probiotics produce several antimicrobial compounds and peptides as a defense mechanism against pathogens [12] but they are not specific. Probiotics can therefore be used as candidates for the production and delivery of therapeutic antimicrobial peptides within the host GIT targeting a specific action or pathogen. The current methods for production of AMPs have been reported to have several limitations. Synthesis of peptides is not only expensive, but also time-consuming too; in some cases, the peptides eventually kill the producing cells or are secreted as inclusion bodies. Oral administration of the peptides subjects them to degradation before they can reach the target site. They are also difficult to administer systemically as they are rapidly identified and directed for restoration of the immune system before they can reach the site of infection. Therefore, an alternative strategy will be to use probiotic strains to express the different AMPs resulting in a combination strategy where hosts will get the probiotic effects with the production of the different AMPs [116].

Volzing et al. [31] chose *L. lactis* as an ideal vehicle for production and delivery of AMPs to the site of GI infection due to its ability to survive within the human gastrointestinal tract and its amenability to heterologous gene overexpression. In their study, they engineered a *L. lactis* strain to inducibly express and secrete AMPs with high activity against Gram-negative pathogens, specifically *E. coli* and *Salmonella* strains. The AMPs of interest, A3APO and alyteserin were selected and then cloned into *L. lactis* for the expression of the heterologous peptides. An expression cassette containing a codon-optimized sequence for alyteserin was fused with an Usp45 secretion signal sequence. This expression cassette was cloned under the control of a nisin inducible promoter and transformed into *L. lactis.* When the resultant *L*. *lactis* recombinant strain was induced to express and secrete these peptides, and the effect of their expression on growth and viability of *E. coli* and *Salmonella* was tested, the results indicated successful inhibition of both these pathogens while viability of the host (i.e. the *L*. *lactis* expressing the peptides) was maintained. Inhibition of these pathogens by alyteserin was observed from concentrations ranging from 0.125–1 mg/ml, while the *L. lactis* strains remained viable when exposed to the alyteserin supernatant at 1 mg/ml. This system showed potential as a therapeutic alternative to antibiotics in order to target and inhibit Gram-negative bacteria.

#### Enhancement of anti-inflammatory response

A group of chronic inflammatory disorders known as inflammatory bowel diseases (IBD) are responsible for the inflammation of the digestive tract. The two forms of the IBD are Crohn’s disease and ulcerative colitis, which are both characterized by an uncontrolled inflammatory response in the intestines [117]. The treatment of IBDs poses a challenge as the current treatment options are either costly or cause severe side-effects in patients. There have been a number of studies on the treatment of IBDs and recent research has reported that probiotic bacteria may counteract the chronic inflammatory process [118]. Elafin, is a protease inhibitor expressed in the intestinal epithelium, which contributes to the reduction of inflammation. During inflammation, there is an increase in elastase and myeloperoxidase (MPO) activity, elafin can inhibit the function of proteases, thereby reducing inflammation [119]. Bermúdez-Humarán et al. [118] bioengineered *L. lactis* to express elafin in mice suffering from colitis. The gene encoding for elafin was fused in frame with a gene encoding for a ribosome-binding site and with an Usp45 secretion signal sequence and inserted into an expression vector. The recombinant plasmid was thereafter transformed into *L. lactis* and expression was induced under the control of a nisin-induced promoter. Colonic inflammation was then induced in mice with dextran sodium sulphate and then the mice were subsequently orally treated with either wild-type or recombinant *L. lactis.* Analysis of mice colons for inflammation parameters such as colonic thickness, elastase activities and granulocyte infiltration after 7 days indicated that mice treated with recombinant *L. lactis* secreting the elafin showed a significant reduction in all inflammation parameters. However, mice treated with wild-type probiotics did not show the same significant decrease in inflammation parameters, their response was similar to that of the control-untreated mice. Furthermore, comparison of efficiency of recombinant *L. lactis* secreting elafin to those expressing either the anti-inflammatory cytokine IL-10 or TGF-β1 (to be discussed next) showed that elafin-secreting strain was the most efficient. These results suggested that elafin was the most efficient anti-inflammatory molecule to be delivered by a probiotic strain at the mucosal surface in order to treat inflammation [118].

Chronic inflammation of IBD patients can also be reduced through the administration of anti-inflammatory cytokines such as interleukin 10 (IL-10). IL-10 plays a central role in down-regulation of inflammatory cascades and in the establishment of tolerance in the mucosa [9]. Interferons (IFN), including IFN-α and IFN-β, are widely expressed cytokines involved in innate responses and additionally, these cytokines have an immunomodulatory role in the anti-inflammatory host response. The use of probiotic bioengineering to treat IBD has been studied and it has been reported that this can indeed be used as an alternative. Several studies have been carried out with regard to probiotics expressing cytokines and other anti-inflammatory molecules such as IL-10 and TGF-β instead of elafin, using similar cloning procedures used for elafin. After transformation, recombinant probiotic strains were induced with nisin in order to either express IL-10 or TGF-β and orally administered to mice suffering from colitis. Recombinant *L. lactis* expressing TGF-β displayed beneficial effects by reducing MPO levels, overall reducing inflammation and colitis in 40% of the mice. However, the protective effects against colitis were higher in mice treated with recombinant probiotics expressing elafin than those treated with probiotics expressing IL-10 [120]. Another study reported that intra-gastric administration of *L. lactis* expressing recombinant IL-10, a cytokine used in clinical trials for treatment of IBD, could successfully prevent colitis in murine models [121].

McFarland et al. [122] investigated the effects of local administration of IFN-β on a murine model of colitis. They developed a transgenic *L. acidophilus* strain that constitutively expresses IFN-β and reported that the resultant recombinant strain secreting IFN-β resulted in the exacerbation of colitis. Tumor necrosis factor α (TNF-α) is a cytokine that mediates the clinical symptoms of IBD [9]. In a study by Vandenbroucke et al. [123], they constructed a recombinant *L. lactis* to produce anti-TNF-α nanobodies and reported that daily administration of this strain reduced the colonic inflammation.

#### Enhancement of colonization exclusion

Enhancement of probiotic adhesion to the intestinal mucosal surface can be seen as a potential strategy in order to prevent adhesion and colonization of pathogenic bacteria. Strategies include using gene products of target pathogens such as adhesins or secretory systems in probiotic bacteria to create a competitive environment for colonization [94]. A number of researchers investigated the efficiency of this approach in improvement of competitive exclusion by enhancing binding or adhesion efficacy of the probiotics to host cells. When internalin A from *L. monocytogenes* was cloned and expressed into the *L. lactis* strain, there was enhanced binding to human epithelial cells and bacterial internalization [124]. A more recent study, Koo et al. [94] developed a recombinant probiotic *L. paracasei* harbouring the *Listeria* adhesion protein (LAP) in order to control *L*. *monocytogenes* infection. LAP interacts with a heat shock protein 60 receptor in host cells and promotes adhesion of *Listeria* to host cells. Conventional and recombinant probiotic *L. paracasei* were added to Caco-2 cell monolayers separately, thereafter these monolayers were Giemsa-stained. Pre-exposure of Caco-2 cell monolayers to recombinant *L*. *paracasei* expressing LAP followed by the addition of *L. monocytogenes* led to a reduction of adhesion and translocation of the pathogen. The wild-type probiotic strain had no significant reduction in the adhesion of the *L. monocytogenes* to the cell monolayer, while the recombinant strain resulted in a 60% reduction of adhesion.

It has been shown that flagellins from *Bacillus cereus* are responsible for the adhesion of the bacteria to mucosal cells [125]. Gut pathogens may also use fimbriae or flagella which are extended appendages on the surface of the cell wall, to adhere to host cell receptors. Therefore, expression of these specific appendages in probiotic strains would allow them to bind to the intestinal epithelium, excluding pathogenic binding. Taking that into consideration, Sánchez et al. [126] cloned the surface-associated flagellin of *B. cereus* CH and expressed it in the probiotic *L. lactis*. The recombinant strain adhered strongly to the mucin-coated polystyrene plates in an in vitro experiment and competitively inhibited the binding and adhesion of pathogenic *E. coli* and *S. enterica*.

Enterotoxigenic *Escherichia coli* (ETEC) K99 fimbriae have been reported to enhance the production of mucosal IgA and serum IgG1 fimbria-specific responses [127], thereby increasing the immune responses at mucosal surfaces such as the gastrointestinal (GI) tract, the respiratory tract, and the vaginal tract [128]. Chu et al. [129] cloned and expressed the K99 fimbriae from ETEC into the probiotic *L. acidophilus* and reported that the recombinant *L. acidophilus* was able to reduce the attachment of ETEC to porcine intestinal brush border in a dose-dependent manner. The reduction of the adherence of the pathogen by the recombinant probiotic prevents the binding of the pathogen, therefore inhibiting the infection.

#### Receptor mimicry system and toxin neutralization

One mechanism that pathogens use to invade the host cells and cause infection is through the production of toxins. These pathogens secrete toxins, and sometimes express adhesins that bind to host cells via oligosaccharide receptors displayed on surface glycolipids or glycoproteins. The interaction between the released toxin and the specific oligosaccharide receptors on the surface of the human intestinal cells is an essential step during pathogenesis [130]. Therefore, toxins or secretory systems of pathogens may also serve as potential targets in development of therapeutics [131]. Taking this into consideration, it thus becomes apparent that interfering with the toxin receptor binding and adhesion can be used as a strategy to exclude the pathogen and subsequently minimize or control its infection [130]. A therapeutic strategy would be to express toxin receptors on the cell surface of probiotic strains in order to mimic the receptor [132]. This expression produces a lipopolysaccharide that mimics a host cell receptor, which, e.g. cholera toxin or ETEC heat-labile toxin could recognize and bind to. Therefore, upon infection, enterotoxins would bind to probiotics and become sequestered, protecting the host from a pathogenic infection [130]. That is, the toxin is sequestered when, instead of binding to the receptor on the surface of the host cell, it binds with high avidity to the receptor mimic expressed on the surface of the probiotic cell. This hinders the interaction between the toxin and the host cells, which is a crucial step in the disease process [130, 134]. Studies of probiotics expressing toxin receptor mimics were mostly biased towards impact on the disease progression without monitoring of the probiotic-toxin complex. However, Paton et al. [130] reported that the receptor mimic probiotic was spontaneously eliminated from the GIT of mice a day or two after the end of its administration. Therefore, more studies tracking the probiotic-toxin complex are required to establish their fate.

There are a number of pathogens that secrete these toxins and among them are, *Vibrio cholerae*, Shiga toxigenic *Escherichia coli* (STEC), ETEC and *Clostridium difficile*, just to name a few. Shiga toxigenic *E. coli* and ETEC both cause enteric infections, they cause gastrointestinal disease and diarrhoeal disease in humans, respectively. If left untreated, these pathogens can cause severe bloody diarrhoea associated with haemorrhagic colitis [133]. In an earlier study by Paton et al. [134], the galactosyl-transferase genes from *Neisseria gonorrhoeae* were cloned and expressed into a non-pathogenic *E. coli*. The results showed that the recombinant *E. coli* was 100% effective in treating mice infected with the normally fatal shiga toxigenic *E. coli*. Then later on in another study, these researchers cloned the glycosyltransferase gene, *Neisseria meningitidis* toxin-specific receptor, into the probiotic *E. coli*, creating a competitive environment for toxin binding to the host cells. Expression of these genes created a cell surface mimic of a shiga toxin receptor. This led to competitive exclusion of the pathogen by the probiotic and subsequently inhibiting its infection. This recombinant strain had a high binding capacity and efficacy in mouse models and was effective in neutralizing shiga toxin variants (stx1 and stx2) [132]. Norton et al. [134] cloned and expressed a tetanus toxin fragment C (TTFC) in *L. lactis.* They then reported that there were increased IgA levels in the host after oral administration of the recombinant probiotic, which led to protection of the host against the infections of the mucous membrane. These results were supported by other studies, where mice immunized with this recombinant probiotic showed more resistance to the lethal challenge with tetanus toxin than those that were not immunized [136, 137].

Pathogens are able to control the expression of their virulence genes by sensing signals from their own species, other bacteria or their environment, a phenomenon termed quorum sensing [12]. Interruption of quorum sensing of the pathogen can be used as an alternative strategy to control the pathogen. Cholera is a life-threatening gastrointestinal infection [138] that is caused by ingestion of water or food (usually undercooked shellfish) contaminated with *V. cholerae* [130]. Following ingestion, *V. cholerae* passes through the stomach, colonizes the small intestine and then release cholera toxin (Ctx), which is responsible for its virulence. It has been hypothesized that neutralization of Ctx in the gut should prevent the disease from developing or at least speed up recovery from an established *V*. *cholerae* infection [130]. The cloning and expression of Ctx receptor into probiotics can therefore be used as an alternative strategy for the treatment of cholera. Focareta et al. [139] constructed a probiotic *E. coli* encoding receptor GM1 (to express the GM_1_ ganglioside) on its surface, which is capable of binding large amounts of Ctx and protecting infant mice from challenge with virulent *V. cholerae*. The resultant recombinant *E. coli* was capable of binding purified Ctx with high avidity and adsorbing >5% of its own weight of toxin in vitro. *V. cholerae* releases cholera autoinducer-1 (CAI-1) and autoinducer-2 (AI-2), which depending of population density, can down- or up-regulate expression of virulence genes [12, 140]. Virulence genes involved are Ctx, which causes diarrhoea, and toxin-coregulated pilus (TCP), which facilitates attachment of vibrios to the intestinal wall. When cell densities are high, expression of genes encoding these virulence factors is reduced, while proteases expressed degrade the attachment matrix with consequent flushing out of the bacterial cells with diarrhoeal fluids. In order to determine the possibility for use of bioengineered probiotic for control of cholera, Duan and March [140] constructed an AI-2 producing *E. coli* Nissle that co-expressed CAI-1. They reported an 80% reduction in Ctx binding to the intestines of mice pretreated with recombinant probiotic, which reduced the chances of infection. These results showed the potential for use of bioengineered *E. coli* Nissle co-expressing CAI-1 and AI-2 for the prevention or treatment of cholera.

#### Vaccination

Probiotics may induce low levels of the immune response. Therefore, probiotics can be bioengineered to deliver immunogenic molecules to the intestinal mucosal surface to enhance the immune response. Recombinant probiotics can act as a vaccine arming the host immune system to deal with gut pathogens [141]. In order to exploit a safe and effective vaccine for the prevention against K99 infections of ETEC, Wen et al. [142] cloned and expressed ETEC adhesins K99 into the probiotic *L. casei*. They reported that there was an increase in the efficacy of the recombinant probiotic and that more than 80% of the vaccinated mice were protected after challenge with a lethal dose of standard strains.

Non-bactericidal infections can be treated with bioengineered probiotics through an approach using vaccination delivery systems. Rotavirus is the most common cause of diarrhoea in children. It damages cells within the small intestine (enterocytes) and thereafter causes gastroenteritis. The viral proteins can disrupt the reabsorption of water within the human intestine and can also cause an inefficiency to digest lactose, resulting in milk intolerance for infants. Symptoms include nausea, vomiting, diarrhoea, fever and dehydration [143]. Gardlik et al. [145] bioengineered *L. lactis* to express virus spike protein VP8, which induced anti-VP8 antibodies and IgA antibodies in mice. This induction occurred systemically and locally within the mouse intestine providing 100% protection against rotavirus. With oral vaccination being favoured above the other types of vaccination, using probiotics with their ability to withstand the GIT conditions can be used as an alternative mode of vaccination. There are several other advantages of delivery of vaccines using recombinant probiotics such as easy administration by consumers, a decreased risk in transmission of foodborne diseases and the stimulation of both innate and adaptive immunity [9].

## Safety concerns regarding bioengineered probiotics

Bioengineered probiotics are increasingly being studied as vehicles that can express and target delivery of specific genes directed towards a specific foodborne pathogen. One of the main drawbacks of working with bioengineered probiotics is that they are classified as genetically modified organisms (GMO) [144]. The nature of such probiotics regarded as GMO presents a major limitation to their widely applications. It is well known that some consumers have ethical reasons for not consuming GMO for fear that such organisms may pose danger to one’s life [145]. Other concerns about GMO relate to their release into the environment and their survival and propagation in this environment, dissemination of antibiotic selection markers or other genetic material to other organisms [146]. Introduction of the GMO into the environment can impact there directly by competing with natural species, or indirectly by changing the balance between native species [147]. However, these modified microorganisms have a great potential to address novel approaches for prevention and treatment of different human and animal pathological conditions. It is therefore, important to establish criteria that can be used for the assessment of the environmental safety and tracing the fate of recombinant DNA in vitro and in vivo, which are both of significant importance [148]. Hence, safety of these strains needs to be guaranteed in order for them not to possess antibiotic selection markers or to transfer genetically modified DNA to other bacteria [144]. Biological containment systems can be used to prevent dissemination of genetic material to other bacteria and to prevent a significant uncontrolled increase of probiotic cells into the natural environment [145]. The organism is genetically programmed to only grow in the laboratory and to die in the natural environment [149]. The use of the thymidine-deficient strains is one of the promising strategies for biological containment of bioengineered probiotics. In these strains, the gene of interest is cloned into the chromosomal thymidylate synthase gene (*thy*A), which codes for production of thymine essential for growth of *L. lactis*. This disruption of the *thy*A gene makes the recombinant strain dependent on external supplementation of thymidine or thymine in the growth medium for growth and survival. Thymine is absent in the environment or its levels are limiting in vivo, and this ensures that the recombinant strain dies rapidly due to the absence of an essential growth component. In addition, chromosomal location of the introduced gene provides stability and reduces the risk of horizontal gene transfer [146, 150].

When cloning and expressing the different virulent traits into probiotics, only traits that will not make the probiotics pathogenic should be used. It is also crucial that each bioengineered strain be carefully evaluated for virulence determinants and sensitivity to clinically relevant antibiotics before being deemed suitable as a probiotic [151]. When cloning probiotics, therapeutic safety of recombinant probiotic carrier organisms is crucial, especially when the strain has to be used in individuals who are already infected with a pathogen. The risk exposure determination, risk assessment and safety assessment are essential to ensure protection for the population against any unintended consequences of the use of probiotics [152].

## Conclusions and future perspective

The rise in morbidity and mortality due to foodborne pathogens remains a serious concern worldwide and the need for an alternative strategy for the control and treatment of infections caused by pathogens is equally crucial. The application of probiotics in food for control of enteric pathogens has been explored and the probiotic market is growing worldwide. The ability of probiotics to inhibit human enteric pathogen has been well researched and documented and this has led to their use as a therapeutic approach for treatment of enteric infections. These studies showed both their successes and limitations, mainly highlighting the generic nature of their mode of action and their failure in controlling some specific pathogens. These limitations can be overcome and functions of conventional probiotics enhanced to create a greater beneficial effect through the use of bioengineering. The modification of conventional probiotics by use of bioengineering technology has a significant potential for design and development of novel therapeutic approaches for effective treatment of pathogens.

Thorough understanding the life cycle of pathogens post ingestion, and knowledge of the virulence factors they use to cause infections offers a strategy for development of bioengineered probiotics strains tailored to control-targeted pathogens. By targeting a specific pathogen, the efficacy of the probiotics inhibiting both the pathogens and infection will be increased. Although still in the early stages, researchers have made impressive strides towards design of such probiotics, producing strains geared towards enhancement of various functional and/or technological probiotic properties. Results from most of such studies showed positive effects although in some cases no benefits were reported. The bioengineered probiotics thus offer important potential to be used as novel therapeutic approach for the prevention and treatment of foodborne infections. More studies targeting different virulence genes and pathogens, including the less studied and emerging ones, are necessary in order to establish the future of this field of research and determine how it will impact on the food and health industries.

In addition to the above, most bioengineered probiotics are designed to be orally administered; therefore, they must still be able to survive through both technological and gastrointestinal stresses. It is also crucial that these strains have scientifically validated health properties, demonstrated safety and good technological properties to be produced on a large scale [147]. They should remain viable in large numbers so as to confer the beneficial effects to the host and should not develop unpleasant flavours or textures upon their incorporation into foods [148]. Furthermore, studies on bioengineered probiotics, specifically for targeted control of pathogens, have focused on the impact of the recombinant probiotic strain on the pathogen(s) of interest. The influence of administration of these probiotics on commensal bacteria or the whole microbiota has not been the subject of studies. These aspects should also be addressed in future studies on bioengineered probiotics.

## Abbreviations

GIT: gastrointestinal tract
LAB: lactic acid bacteria
IgA: immunoglobulin A
IL-10: interleukin 10
RBC: ranitidine bismuth citrate
BetL: listerial betaine uptake system
AMPs: antimicrobial peptides
MDR: multi-drug-resistant
IBD: inflammatory bowel diseases
MPO: myeloperoxidase
LAP: *Listeria* adhesion protein
ETEC: enterotoxigenic *Escherichia coli*
STEC: Shiga toxigenic *Escherichia coli*
TTFC: tetanus toxin fragment C
GMO: genetically modified organisms
TCP: toxin coregulated pilus
*thy*A: thymidylate synthase gene
